# The Ideal Physical Therapist from the Perspective of Individuals With Limb Loss

**DOI:** 10.33137/cpoj.v6i1.42093

**Published:** 2023-12-22

**Authors:** D.J Lee, A Gambale, M Nisani, C Miller, E Leung, M Rodgers, D Chilianis, M Marra

**Affiliations:** 1 Department of Physical Therapy, Stony Brook University, Stony Brook, NY, USA.; 2 Department of Physical Therapy, Philadelphia College of Osteopathic Medicine, GA, USA.

**Keywords:** Physical Therapy, Therapeutic Alliance, Limb Loss, Amputation, Prosthesis, Rehabilitation

## Abstract

**BACKGROUND:**

Effective rehabilitation after limb loss is necessary to maximize function and promote independence. Physical therapists (PT) are one of the primary drivers of the rehabilitation process. While general physical therapy knowledge and abilities have been shown to be important to the rehabilitation process, it is unclear what individuals with limb loss value in their PT's.

**OBJECTIVE:**

The purpose of this study was to understand the elements that define an ideal PT from the perspective of individuals with limb loss.

**METHODOLOGY:**

Mixed-method design consisting of a 20-item web-based survey and semi-structured interviews that were administered to individuals 18 years or older, who spoke English, and had a history of lower limb loss.

**FINDINGS:**

Individuals with limb loss describe an ideal PT as promoting a therapeutic alliance, having specialized knowledge, and collaborating with a prosthetist. Knowledge of the PT as it relates to limb loss was found to be both the greatest facilitator and barrier to the rehabilitation process.

**CONCLUSION:**

From the perspective of those with limb loss, an ideal PT promotes a strong therapeutic alliance through communication, has specialized knowledge when it comes to the limb loss rehabilitation process, and collaborates with the prosthetist to problem-solve throughout the rehabilitation process.

## INTRODUCTION

With approximately two million individuals living with limb loss in the United States, it is paramount that individuals with limb loss receive appropriate treatment following this life altering event.^1,2^ Limb loss can impact an individual's quality of life and function by limiting participation in valued activities. However, a comprehensive individualized rehabilitation program can help build a functional foundation for a safe transition back to society and daily life for individuals with limb loss.

The rehabilitation team is multidisciplinary in nature and includes prosthetists and Physical therapists (PT) as two key members. While the prosthetist has domain over the design, delivery, and function of the prosthetic device, the PT's are responsible for promoting optimal physical function after the loss of a limb.^3^ Research has shown the importance of the collaborative decision-making process between prosthetists and individuals with limb loss and the positive impact it has on patient-centered outcomes.^4^ This synergistic process was echoed for PT's, with collaborative goal setting and problem-solving being of importance to the rehabilitation process.^5^

While qualitative studies have explored what those with limb loss value in the rehabilitation process and in their relationship with the prosthetist, there is little evidence on what traits make an ideal PT. A PT's capacity to understand the qualities in which persons with limb loss value during rehabilitation may open the door for more efficacious treatment and improved outcomes. To our knowledge, there is a void in published research examining the traits and skill set of a PT that individuals with limb loss deem valuable. Therefore, the purpose of this study is to understand the elements that define an ideal PT from the perspective of persons with limb loss.

## METHODOLOGY

***Study Design:*** This study utilized a mixed methods design following the COREQ checklist for qualitative data collection.

***Setting:*** Amputee Coalition National Conference and video conferencing.

***Participants:*** Inclusion criteria were individuals who attended the Amputee Coalition National Conference (August 2020), were 18 years of age or older, spoke English, had a history of lower limb loss with or without prosthesis use, and performed physical therapy after loss of limb. Exclusion criteria included individuals that demonstrated cognitive impairments consistent with an inability to complete the survey and/or interview.

***Ethics:*** Institutional Review Board (IRB) approval was obtained prior to the collection of data. All participants completed the informed consent process prior to starting the study.

***Instruments:*** The quantitative portion of the study utilized a 20-item web-based survey hosted by QualtricsXM (Provo, UT) designed specifically for use in this study. The survey can be found in **Appendix A**. The qualitative portion of the study was performed using a semi-structured interview that explored participants feelings and beliefs about physical therapy care. The interview questions can be found in **Appendix B**. Prior to data collection, the survey and interview were piloted for ease of use, logic functionality, and efficiency, then revised based on feedback. Researchers were trained in interview skills and procedures by an expert in mixed-methodology, completing two pilot interviews prior to data collection. Participants provided written and/or verbal consent prior to participating in the survey and semi-structured interviews.

***Procedures:*** All data were collected remotely using web-based surveys and video conference interviews secondary to social distancing mandates present at the time of the data collection (August 2020). Participants who attended the Amputee Coalition National Conference had the ability to visit the researcher's virtual booth to learn about the survey and interview. An anonymous link was provided to the survey on a display page linked to the virtual booth. If a participant additionally wanted to participate in an interview following the survey, they were eligible for a $10 digital gift card.

Interviews were conducted via video conferencing software. There were no relationships established between the participants and researchers prior to the study commencement. Each interview was audio recorded and transcribed prior to data analysis.

**Data Analysis:** Quantitative data were analyzed for trends using SPSS v25 (IBM Corp. Aramonk, NY). Identified trends were presented in terms of means and likert scales ranked by participants. Qualitative data were analyzed using an inductive approach with a constant comparison method to identify themes. The process was multi-stage, involving a pair of researchers first independently analyzing the transcripts from the interview to generate codes. Then each member of the pair would compare their codes and work towards consensus. Each pair analyzed four to five unique transcripts. Once all the transcripts were coded, all research team members compared the generated codes and revised for consistency using the consensus method. Once the codes were agreed upon, themes were then generated using the same iterative process described for the codes. Disagreement was adjudicated by a separate researcher not involved in the original coding.

Sample size calculations were not performed for the survey portion given its descriptive nature and lack of statistical comparisons requiring adequate power to yield significance. However, an incentive of a $10 gift card was provided to encourage participation in the study as the greater number of data points allows for a more comprehensive understanding of the results. The qualitative sample size is based on the principle of data saturation which supports a smaller sample (typically 10–15 participants) for homogenous groups (e.g. individuals with lower limb loss who have experienced physical therapy attending a national conference).

## RESULTS

### Quantitative

#### Characteristics of Study Population

Of the 92 completed surveys, 22 were excluded due to failure to provide complete demographic information, leaving 73 completed surveys. Incomplete surveys were excluded to reduce potential bias and provide a more accurate representation of the study population. The participants were primarily female (53.0%) with a mean age of 54.4 years. Most participants were educated beyond high school (88.0%), had an average of 13.6 years since their initial amputation, with most having had a transtibial level amputation (51%) due to trauma or infection (54.0%). Predominant comorbidities of hypertension (29.7%) and hypercholesterolemia (14.9%) were most common. A summary of the demographic characteristics is presented in **Table 1**.

**Table 1:** Survey Demographics.

Demographics of study population (n =73)	
**Gender:**	%
• Female	53
• Male	47
**Age: (Mean ± SD)**	54.4 ± 16.2
**Highest Level of Education:**	%
• General Educational Development Test	1
• High School	11
• College	58
• Post-Graduate	30
**Years Since 1^st^ Amputation: (Mean ± SD)**	13.6 ± 14.9
**Reason for 1^st^ Amputation:**	%
• Trauma	31.1
• Infection	23.0
• Congenital	10.8
• Tumor	14.9
• Vascular Disease	13.5
• Other	6.8
**Current Amputation Level:**	%
• Transtibial	51
• Transfemoral	44
• Hip disarticulation	4
• Foot	1
**Medical History:**	%
• Diabetes	14.9
• High blood pressure	29.7
• High cholesterol	17.8
• Arthritis	9.9
• Heart Disease	10.9
• COVID-19	2.0
• Other	14.9

#### Barriers to Physical Therapy

**Figure 1:**
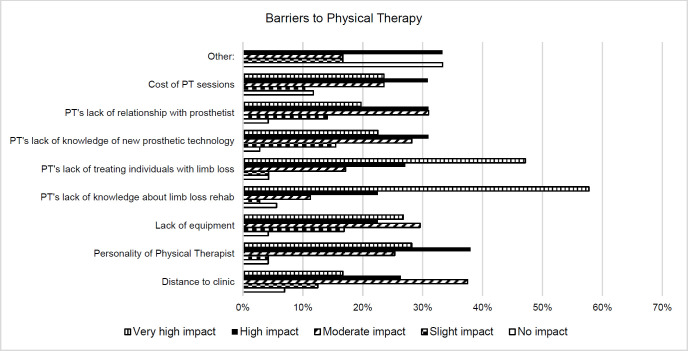
Barriers to Physical Therapy.

Responses from participants reported the PT's lack of knowledge about limb loss (58%) and PT's lack experience treating persons with limb loss (47%) as having a very high impact as a barrier to physical therapy. Additionally, the personality of the PT (38%) was reported to have a high impact as a barrier to physical therapy. Meanwhile, the cost of the physical therapy sessions (12%) and distance to the clinic (7%) were most frequently regarded as having no impact as a barrier to treatment. Responses are shown in **Figure 1**.

#### Facilitators to Physical Therapy

**Figure 2:**
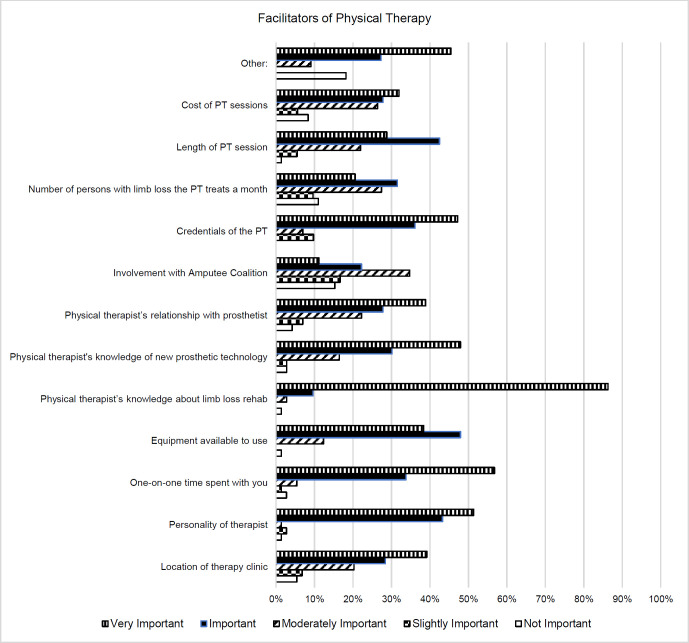
Facilitators to Physical Therapy.

Participants reported a PT's knowledge about limb loss rehabilitation (86%), one-on-one time spent with PT (57%), and the personality of the PT (51%) serving as very important facilitators of physical therapy. Available equipment was most frequently (48%) regarded as an important facilitator of physical therapy. Participants regarded involvement with Amputee Coalition (15%), number of patients with limb loss the PT treats a month (11%), and cost of physical therapy session (8%) as having no importance as a facilitator of physical therapy. Responses are shown in **Figure 2**.

#### Knowledge of the PT

Responses from participants reported knowledge of gait training with prosthesis (74%), knowledge of prosthesis fit (63%), and knowledge of when to contact a prosthetist (62%) as being very important. Knowledge of current research regarding limb loss (35%) was most frequently regarded as being important. Meanwhile, knowledge of prosthetic components (10%) and knowledge of problem-solving issues with fit of prosthesis (9%) were most frequently reported as having slight or no importance.

#### Physical Therapy Experience

**Figure 3:**
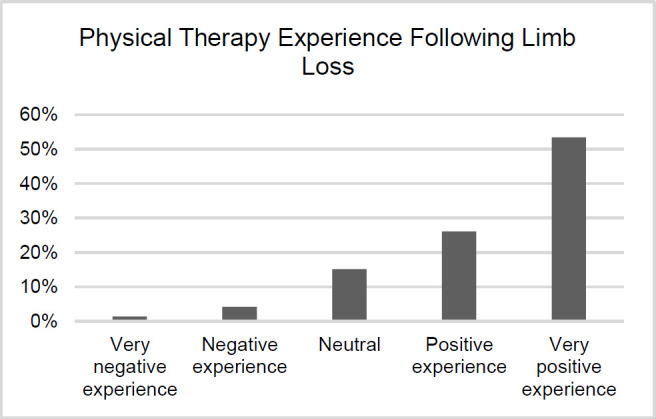
Physical therapy Experience Following Limb Loss.

Participants (79%) regarded having a very positive or positive experience with physical therapy, with 53% of participants reporting having a very positive experience. Responses are shown in **Figure 3**.

### Qualitative

Of the 73 completed surveys, 13 participants agreed to participate in conducted interviews. A summary of the demographic characteristics is presented in **Table 2**. Three themes were identified from the interviews:

**Table 2:** Interview Demographics.

**Demographics (n=13)**	
**Gender:**	%
Female	77
Male	23
**Age: (Mean ± SD)**	54.5 ± 14.7
**Highest Level of Education:**	%
GED	0
High School	7.7
College	61.5
Post-Graduate	30.8
**Years Since 1^st^ Amputation: (Mean ± SD)**	9.9 ± 9.5
**Reason for 1^st^ Amputation:**	%
Trauma	30.8
Infection	23.1
Congenital	0
Tumor	15.4
Vascular Disease	7.7
Other	23.1
**Current Amputation Level:**	%
Below knee	54
Above knee	46
Hip disarticulation	0
Foot	0
**Medical History:**	%
Diabetes	18.8
High blood pressure	18.8
High cholesterol	18.8
Arthritis	6.3
Heart Disease	12.5
COVID-19	0
Other	25

#### Theme 1: Communication is Key

Participants reported valuing the therapeutic alliance that forms between the individual with limb loss and the PT. Communication, an element of the therapeutic alliance, was cited as either a facilitator or barrier, as expressed in the participant quotes below:

*“The ones I hold in the highest esteem are the ones that… one, listened to me and recognized things that I needed to do day in and day out in terms of whatever my job was.”*

*“It's got to be a relationship built with communication, where the therapist has to listen to what the patient is and isn't saying in response to things they are asked to do.”*

*“When the PT listens to what I'm asking and understands or listens and reacts to what I need; not necessarily what a traditional rehabilitation program is. Because I think it needs to be really individualized.”*

*“I got so frustrated with the PT because I didn't need it… I wanted her to help me walk but she had me& every time I came, take off my prosthetic and do exercises. This is not why I'm here. I want to get on a treadmill. I want you to tell me how to walk because I'm walking with a limp*.”

From these quotes it is evident that positive communication was supportive of a healthy therapeutic relation, while lack of clear communication, as demonstrated in the last quote, can lead to frustration, which in turn can weaken the therapeutic alliance.

#### Theme 2: Specialized Care for a Specialized Population

Participants reported that specialized knowledge was one of the most significant components of high-quality therapy for individuals with lower limb loss. Participants in the study emphasized:

*“I think it's important for amputees to work with someone who has an expertise in amputees and prosthetics, because it is such a specific niche in the field.”*

*“It's important that the PT has knowledge of the different prosthetics and the features, because if they don't know it, I probably don't either!”*

*“First of all, a PT who is trained in the nuances of working with an amputee, that's a must. Because not only do we not ambulate the same, but we don't have the ability to do many of the normal limb functions.”*

*“The reason he [the PT] knew my leg was too short was he had different samples of different thickness socks. What he would have me do is have me stand on different ones, different thicknesses, until he determined this is the correct height. They didn't do that in the large rehab hospital. That's that extra attention to detail, his experience and knowledge gave him.”*

The theme outlines how individuals with limb loss value a PT with specialized knowledge and equipment. A PT with more knowledge and an in-depth understanding of limb loss rehabilitation is considered a more desirable provider for persons with limb loss.

#### Theme 3: The Dynamic Duo: PT's and Prosthetists

Participants in this study reported an increase in quality of care when the prosthetist was an active collaborator in their physical therapy plan of care. For instance, several participants found it beneficial to have the prosthetist present at their treatment sessions due to their expertise in handling prosthetic limbs and deeper comprehension of the mechanical nature of these medical supportive devices. Perceptions of an effective therapeutic team were reiterated by participants:

*“My prosthetist was at my physical therapy appointments. She could adjust it while I was actually with a PT, and she could see what was happening while I was doing it. That made it a very positive experience.”*

*“They [the PT's] also bring in the certified prosthetist. That prosthetist is going to know more about that particular prosthetic that individual is wearing than the PT themselves.”*

*“I found that the prosthetists were really the ones that know the devices much better, in order to gait train properly you really have to understand the mechanics of the device.”*

To provide the most comprehensive care for a patient with limb loss, it is crucial for the PT to recognize the strength in the specialized knowledge possessed by a prosthetist and the integral role they can play in facilitating increased quality patient care. When asked to describe what makes a PT ideal for working with persons with limb loss, participants described:

*“Being open to the prosthetist, being open to the knowledgeable amputees that are out there and learning from them and then being able to share your expertise.”*

In summary, incorporating the prosthetist benefits both the patient and the therapist, and therefore should be part of the treatment sessions and goal setting.

## DISCUSSION

The purpose of this study was to explore the elements that define an ideal PT from the perspective of individuals with limb loss. The results of this study demonstrated that individuals with limb loss value the PTs knowledge regarding limb loss rehabilitation, the collaboration between the prosthetist and the PT, and a strong therapeutic alliance.

### The Therapeutic Alliance

The therapeutic alliance defines a shared trust and collaboration between the patient and the healthcare practitioner,^6^ and can be promoted through collaboration, communication, and personalized care for individuals with limb loss.^4,7^ The core of the therapeutic alliance is a patient-centered approach, a finding that is evident from the qualitative portion of this study. For example, patients who are engaged in decision-making for cosmetic design of the prosthesis felt empowered and had greater satisfaction with their prosthesis.^7^ While placing value and emphasis on building interpersonal relationships that foster healing has been found to be as important to outcomes as chosen interventions in physical therapy.^8^ Additionally, health professionals that express their understanding and empathy with the focus on the patient, increase patient trust and contribute to positive experiences with physical therapy and prosthetic management.^4,9^ These findings are consistent with our study where participants related the importance of collaboration as a means to support healthy therapeutic relationships.

Participants in this study noted that communication can also be a barrier or facilitator to the rehabilitation process depending on the quality of the interaction. Those participants who had a positive experience described the communication to be purposeful and focused on their specific needs as an individual with limb loss. However, at least one participant noted that, despite best intentions by the PT, the attempts at communicating were contributing to a negative experience because of the lack of collaborative goal-setting.

Active engagement and tailored communication related to decision-making and goal setting has been reported to enhance patient satisfaction with musculoskeletal physical therapy across settings and in private practice.^6,10,11^ Findings from this study, corroborates results in the field of limb loss rehab and with other populations seen in physical therapy settings, further illustrating that strong therapeutic alliance built on positive communication leads to better outcomes.^8,10^-^12^

### Specialization

Of interest in this study, there was a disconnect between some of the participants quantitative versus qualitative values on the PT's knowledge of prosthetic devices. However, Shih et al.^12^ found that individuals who received physical therapy expressed more positive comments related to perceived positive outcomes and benefits of therapy rather than on the prosthetic design. Given that technological changes are abundant in the field of prosthetics, it would seem valuable for the PT to have high levels of knowledge about the prosthetic devices and their components. While this specialized knowledge of devices was promoted in some of the qualitative results, we saw less importance placed on this knowledge area than gait training or being able to manage the fit of the prosthesis.

In the United States, less than 50% of the PT programs have stand-alone courses that spend a considerable amount of time promoting prosthetic-related content in their programs specific to the devices and components themselves.^13^ Additionally, there is tremendous variability in the content emphasized and educational hours dedicated to training physical therapist ostensibly for reasons related to patient-centered outcomes. This may be interpreted as individuals with limb loss valuing the functional mobility and problem-solving aspects of patient care more than explicit knowledge on the prosthetic components.

While components and devices were not regarded as being highly important, generally, a therapist's knowledge levels were shown to be either a facilitator or barrier to the rehabilitation process. While the mixed findings from this study do not clearly suggest knowledge of devices alone is of significant importance to the person with limb loss, it does emphasize the importance of being knowledgeable generally about the process in order to facilitate positive outcomes and reduce unwanted secondary complications.^14^

### Collaboration with the Prosthetist

Building off the ideas of therapeutic alliances and the need for specialization, participants in this study valued the collaboration between the prosthetist and PT. Coordinated care with improved pathways for access to amputation management, including PT, prosthetists, and peer-visitors was identified as essential for timely recover in individuals with limb loss.^15^ Since the prosthesis is a medical device, alterations to its alignment and function are typically outside of the scope of a PT. This can create a frustrating experience during the rehabilitation process when modifications are needed but the prosthetist is not present. Therefore, expressed frustration as reported in our study may be mitigated by having coordinated sessions where the PT and prosthetist work together to problem-solve gait and functional issues, with the prosthetist performing real-time modifications during the treatment. The ideal outcome is that expedient solutions to mobility issues are provided without delaying the progression of the rehabilitation process.

### Limitations

As is the nature of qualitative research, the biases and beliefs of the researchers themselves may influence the interpretation of the data. While the research team employed techniques like triangulation, internal auditing, and independent adjudication to minimize the influence of bias, the fact that all members of the team were PT's is worth noting. While we do not believe the data and results were influenced by this, we as researchers acknowledge that our view of physical therapy is positive and found the results to be confirmatory of these beliefs. Another limitation is the generalizability of these results outside of the participant pool present at the Amputee Coalition National Conference. While demographically they are representative of individuals with limb loss, the small sample size limits the generalizability of the results, requiring larger scale studies for future research.

## CONCLUSION

From the perspective of those with limb loss, an ideal PT promotes a strong therapeutic alliance through communication, has specialized knowledge when it comes to the limb loss rehabilitation process, and collaborates with the prosthetist to problem-solve throughout the rehabilitation process.

## DECLARATION OF CONFLICTING INTERESTS

None.

## AUTHOR CONTRIBUTION

- **Daniel J. Lee:** Contributed to and oversaw all aspects of the study.
- **Albert Gambale:** Contributed to data collection, analysis, and writing of the manuscript.
- **Maya Nisani:** Contributed to data collection, analysis, and writing of the manuscript.
- **Carol Miller:** Contributed to review and interpretation of results and writing of the manuscript.
- **Elizabeth Leung:** Contributed to data collection and analysis.
- **Madeline Rodgers:** Contributed to data collection and analysis.
- **Daniel Chilianis:** Contributed to data collection and analysis.
- **Matthew Marra:** Contributed to data collection and analysis.

## SOURCES OF SUPPORT

No sources of support to report.

## ETHICAL APPROVAL

Institutional Review Board (IRB) approval was obtained prior to the collection of data. All participants completed the informed consent process prior to starting the study.

## APPENDIX A

- Rate your physical therapy experiences (overall): 0–5 (5 is the highest)
- Rate and rank factors that influence your physical therapy experience
  - Location of therapy clinic
  - Personality of therapist
  - One-on-one time spent with you
  - Equipment available to use
  - PT's knowledge about limb loss rehab
  - PT's knowledge of new prosthetic technology
  - PT's relationship with prosthetist
  - Involvement with Amputee Coalition
  - Credentials of the PT
  - Number of persons with limb loss PT treats a month
  - Specialized equipment
  - Length of PT session
  - Home program
  - Cost of PT
  - Other: ________________
- Rate and rank barriers to physical therapy
  - Distance to clinic
  - Transportation to the clinic
  - PT's' personality
  - Lack of equipment
  - PT's lack of knowledge about limb loss rehab
  - PT's lack of treating amps
  - PT's lack of knowledge about new prosthetic technology
  - PT's lack of relationship with prosthetist
  - Cost of PT
  - Other: ______________
- Rate and rank the importance of the PT's knowledge
  - Knowledge of the prosthetic components
  - Knowledge of gait training with a prosthesis
  - Knowledge about donning and fitting the prosthesis
  - Knowledge about problem-solving issues with the fit of the prosthesis
  - Knowledge of knowing when to contact prosthetist
  - Knowledge of current research regarding limb loss
- Do you believe you would have benefited from more, less, or the same amount of physical therapy rehabilitation?

## APPENDIX B

1. What is/was your experience with physical therapy in regard to your limb loss?
2. In your opinion, what made or makes a physical therapy experience positive for you?
3. In your opinion, what made or makes a physical therapy experience negative for you?
4. What are ways you believe physical therapy can be improved for persons with limb loss?
5. Can you describe any things that your PT does or says that make them “ideal” for working with persons with limb loss?
